# Blood Pressure and Arterial Stiffness in Kenyan Adolescents With the Sickle Cell Trait

**DOI:** 10.1093/aje/kwx232

**Published:** 2017-06-12

**Authors:** Anthony O Etyang, Christopher K Wandabwa, Sailoki Kapesa, Esther Muthumbi, Emily Odipo, Marylene Wamukoya, Nicholas Ngomi, Tilahun Haregu, Catherine Kyobutungi, Thomas N Williams, Johnstone Makale, Alex Macharia, J Kennedy Cruickshank, Liam Smeeth, J Anthony G Scott

**Affiliations:** 1 KEMRI Wellcome Trust Research Programme, Kilifi, Kenya; 2 London School of Hygiene and Tropical Medicine, London, United Kingdom; 3 African Population and Health Research Center, Nairobi, Kenya; 4 Imperial College London, London, United Kingdom; 5 King’s College London, London, United Kingdom

**Keywords:** arterial stiffness, blood pressure, hypertension, sickle cell trait

## Abstract

The potential association between sickle cell trait (SCT) and increased arterial stiffness/blood pressure (BP) has not been evaluated in detail despite its association with stroke, sudden death, and renal disease. We performed 24-hour ambulatory BP monitoring and arterial stiffness measurements in adolescents raised in a malaria-free environment in Kenya. Between December 2015 and June 2016, 938 randomly selected adolescents (ages 11–17 years) who had been continuous residents of Nairobi from birth were invited to participate in the study. Standard clinic BP measurement was performed, followed by 24-hour ambulatory BP monitoring and arterial stiffness measurement using an Arteriograph24 (TensioMed Ltd., Budapest, Hungary) device. SCT status was determined using DNA genotyping in contemporaneously collected blood samples. Of the 938 adolescents invited to participate, 609 (65%) provided complete data for analysis. SCT was present in 103 (15%). Mean 24-hour systolic and diastolic BPs were 116 (standard deviation (SD), 11.5) mm Hg and 64 (SD, 7) mm Hg, respectively, in children with SCT and 117 (SD, 11.4) mm Hg and 64 (SD, 6.8) mm Hg, respectively, in non-SCT children. Mean pulse wave velocity (PWV) was 7.1 (SD, 0.8) m/second and 7.0 (SD, 0.8) m/second in SCT and non-SCT children, respectively. We observed no differences in PWV or in any clinic or ambulatory BP-derived measures between adolescents with and without SCT. These data suggest that SCT does not independently influence BP or PWV.

The sickle cell trait (SCT), common among populations of African descent (1) due to the protection it offers against malaria (2–4), has been associated with increased risk of cardiovascular and renal disease (5–8). However, the underlying mechanisms of this increased risk have not been elucidated clearly, hampering measures that can be implemented to reduce risk in carriers.

Persons of African descent have relatively higher blood pressures (BPs) than other ethnic groups (9), and it is conceivable that increased BP and/or arterial stiffness, which have been shown to precede clinical events similar to those seen in persons with SCT, could precede the events observed in SCT carriers (8, 10, 11). Alternatively, increased arterial stiffness and BP in persons with SCT could result from the yet-to-be-elucidated mechanisms that lead to cardiovascular and renal events.

Previous studies that assessed BP and/or arterial stiffness in persons with SCT had several weaknesses. Rossi-Espagnet et al. (12), in a study conducted in Colombia in 1968, found no difference in BP between persons with and without SCT. However, BP measurements were performed only once at home visits; there was a poor response rate in men; and the data were susceptible to confounding by malaria, which SCT protects against (2–4) and which is possibly related to BP (13, 14). Bayramoğlu et al. (15) found similar arterial stiffness indices in young Turkish adults with and without SCT, but the sample size was small. Although the studies demonstrating increased cardiovascular and renal disease risk in SCT assessed BP at baseline, the BP measurements were taken in the clinic/office, using manual or automated methods (7, 8). None of these studies utilized 24-hour ambulatory blood pressure monitoring (ABPM), which is considered the reference method for BP measurement (16, 17), raising the possibility that subtle but significant differences in BP could have been missed (17). ABPM overcomes many of the limitations of office/clinic BP measurement (17). ABPM also enables detection of masked hypertension (normal clinic BP but elevated 24-hour BP), a cardiovascular risk factor (18) that is more common in populations of African descent (19)—the same population that has a high prevalence of SCT.

If arterial stiffness and/or BP are increased in young persons with SCT, they could become the target of interventions aimed at reducing future cardiovascular and renal events. We conducted a population-based study in Nairobi, Kenya, to determine whether SCT influences arterial stiffness and BP among adolescents who have had minimal exposure to malaria.

## METHODS

The study was conducted from December 2015 to June 2016 in a cross-sectional sample of residents of the area covered by the Nairobi Urban Health and Demographic Surveillance System (20). The area has a population of approximately 70,000, and the prevalence of hypertension is high (21). Nairobi, the capital city of Kenya, was chosen for this study for 2 reasons. First, Nairobi is located at a high altitude (1,800 m above sea level), and there is no evidence of malaria transmission there (22). This made it possible to study the association of SCT with BP unconfounded by the presence of malaria. This was necessary because malaria could influence BP (14), and at the same time SCT protects against malaria (3, 4). Second, the population of Nairobi is composed of ethnic groups originating in all parts of the country, including those whose ancestral lands were endemic for malaria (e.g., Luhya, Luo, Teso, Mijikenda). The frequency of the sickle cell gene is much higher among these ethnic groups (23). In order to increase our efficiency in recruiting participants with SCT, we limited our recruitment to those who identified themselves as genetically descended from one of these ethnic groups.

Population-wide censuses are conducted 4 times a year within the study area (20). Using census data, we selected all children currently aged 11–17 years who had a continuous record of residence in the area since birth. Continuous residency was a requirement so as to minimize potential exposure to malaria as a result of migration. Trained staff visited all subjects who had been selected to participate in the study at their homes. Parents of the children were then asked to bring them to the nearer of 2 study clinics within the area to undergo study procedures. Subjects who failed to come to the clinic within 3 months of being requested to do so were considered to have refused to participate in the study.

Subjects and/or their parents first underwent an interview in which they answered questions about their medical history and their socioeconomic status, based on the multidimensional poverty index (24). Weight and height were measured using a validated Seca 874 flat scale and a portable stadiometer (Seca 213) (Seca GmbH & Co. KG, Hamburg, Germany), respectively. Mid-upper arm circumference was measured in a standardized manner using Teaching-aids at Low Cost (TALC) tapes (Health Books International, Hertfordshire, United Kingdom). We then took a screening BP measurement using a validated automated Omron M10-IT BP monitor (Omron Healthcare Europe B.V., Hoofddorp, the Netherlands). An appropriate-size cuff was placed on the nondominant arm after the subject had been seated for at least 5 minutes. Three BP measurements were taken over a 5-minute period, and the mean of the last 2 measurements was recorded as the screening BP value. All participants were subsequently fitted with an Arteriograph24 device (TensioMed Ltd., Budapest, Hungary) for 24-hour ABPM and determination of pulse wave velocity (PWV) (25). The devices, which have been calibrated in children (26), were programmed to take measurements every 20 minutes during daytime hours (06:00–22:00 hours (6 am–10 pm)) and every 40 minutes at night (22:00–06:00 hours (10 pm–6 am)).

### Laboratory procedures

We collected 10 mL of blood from participants for a full blood count, determination of sickle hemoglobin status, and measurement of serum electrolytes. After automated full blood counts were performed using an AcT 5 diff AL Hematology Analyzer (Beckman Coulter, Inc., Brea, California), whole blood samples were frozen at -80°C and then transported to the KEMRI Wellcome Trust Research Programme laboratories in Kilifi, Kenya, for determination of sickle hemoglobin status. DNA was extracted retrospectively from the frozen samples by use of QIAamp DNA Blood Mini Kits (QIAGEN Ltd., Crawley, United Kingdom) and typed for sickle hemoglobin using polymerase chain reaction.

Serum and urine samples collected from participants were frozen at −80°C within 4 hours of collection and later transported to Kilifi for analysis. We determined levels of sodium, potassium, urea, and creatinine in these samples using ion electrophoresis and the Jaffe method, respectively (27). We additionally determined albumin levels in the urine samples by immunoturbidometry using a Quantex microalbumin kit (Instrumentation Laboratory, Barcelona, Spain).

### Statistical methods

Based on an expected minimum prevalence of 10% SCT in the ethnic groups we were studying, a systolic BP standard deviation of 15 mm Hg, and 30% attrition due to poor-quality ABPM data, we estimated that a total of 550 participants would provide 80% power to detect a 0.5-standard-deviation (7.5-mm Hg) difference in 24-hour systolic BP between children with and without SCT.

Because there are no published criteria for acceptable ABPM data in children, we used guidelines for completeness of ABPM data in adults from the International Database of Ambulatory Blood Pressure in Relation to Cardiovascular Outcome (IDACO) Study (28). Specifically, ABPM data were considered to be of acceptable quality if they met the following criteria: minimum of 10 daytime readings and minimum of 5 nighttime readings, where day was defined as 10:00–20:00 hours (10 am–8 pm) and night as 00:00–06:00 hours (12 am–6 am) (28). The same time periods were used to determine average daytime and nighttime BPs and to evaluate dipping status. Time weighting was applied in calculating average BP values for all time periods (29).

We defined children screening positive for hypertension as those for whom the mean of the last 2 clinic BP measurements was above the 95th percentile for their age, sex, and height (16). Children with confirmed hypertension were those whose 24-hour average systolic and/or diastolic BPs were above the 95th percentile for their sex, age, and height (16).

Using the combination of clinic BP measurements and ABPM, we categorized all subjects who were not on antihypertensive medication into 4 categories: sustained hypertensives (screen-positive and confirmed hypertensive on ABPM); white-coat hypertensives (screen-positive, not confirmed hypertensive on ABPM); masked hypertensives (screen-negative, confirmed hypertensive on ABPM); and normotensives (screen-negative, not confirmed hypertensive on ABPM) (30).

Nocturnal blood pressure dipping was defined using ABPM data only, using day and night periods as defined above. Subjects were classified using the following 4 categories, based on the night/day ratio of mean systolic and/or diastolic BPs: rising or absence of dipping (ratio ≥1.0); mild dipping (0.9 < ratio ≤ 1.0); dipping (0.8 < ratio ≤ 0.9); and extreme dipping (ratio ≤0.8) (31).

Estimated glomerular filtration rate (eGFR) was calculated using the Schwartz formula (32).

Summary statistics computed included mean values, median values, and proportions as appropriate. Comparisons between SCT carriers and noncarriers were made using Student’s *t* test and χ^2^ tests as appropriate. Data that were not normally distributed were log-transformed prior to analysis. We compared ABPM and arterial stiffness measures between children with and without the SCT by Student’s *t* test. In addition, we performed a multivariate regression analysis testing the association of SCT carrier status with mean 24-hour systolic and diastolic BP, with sex, age, body mass index, PWV, and eGFR included as covariates.

All analyses were conducted using Stata software, version 12 (StataCorp LP, College Station, Texas).

The Ethical Review Committee of the Kenya Medical Research Institute (KEMRI) approved the study. Written informed consent was obtained from the parents of study participants. Participating children also provided written assent.

## RESULTS

**Figure 1.**
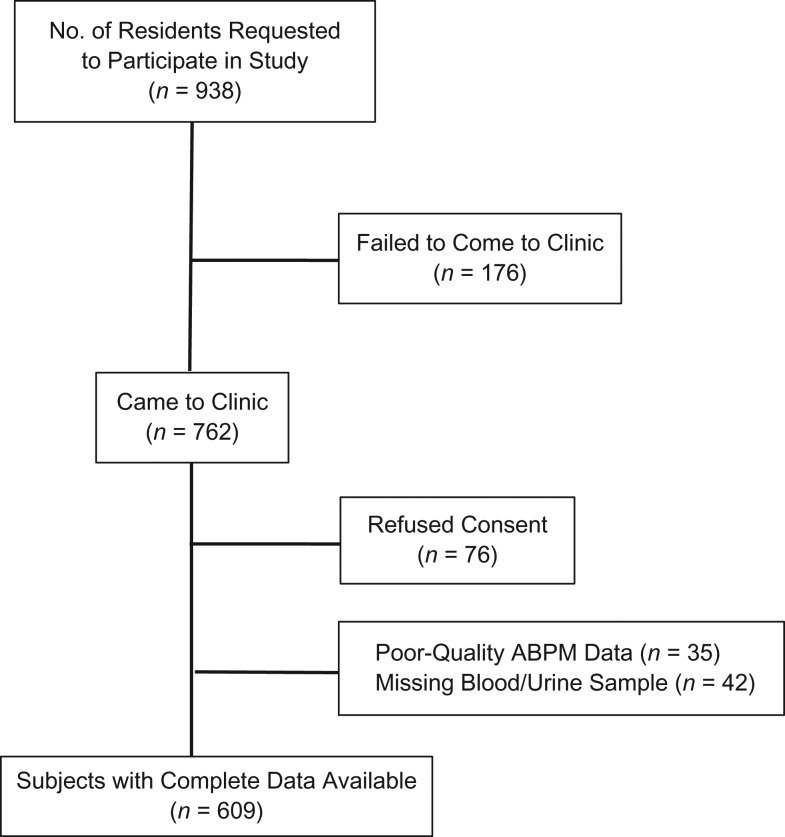
Selection of adolescent participants (ages 11–17 years) for a study of the association between sickle cell trait and blood pressure, Nairobi, Kenya, 2015–2016. ABPM, ambulatory blood pressure monitoring.

Of the 938 subjects asked to participate in the study, 686 (73%) completed enrollment (Figure 1). The 252 adolescents who were not recruited into the study were 0.6 years (95% confidence interval (CI): 0.3, 0.9) older than study participants but had a similar sex distribution (53% female) as those who participated in the study. Genotype data were available for 644 subjects, 609 (95%) of whom had complete ABPM data. SCT was present in 103 participants (15%). The proportion of participants with complete ABPM data did not differ by SCT status (91% vs. 90%; *P* = 0.817) or any of the other demographic and clinical data collected. One participant had sickle cell disease (hemoglobin SS) and was dropped from analyses. None of the participants were previously aware of their SCT carrier status.

Mean systolic and diastolic clinic BPs among all participants were 98 (standard deviation (SD), 11) mm Hg and 64 (SD, 8) mm Hg, respectively. The mean 24-hour systolic and diastolic BPs for all participants were 117 (SD, 11) mm Hg and 64 (SD, 7) mm Hg, respectively. Mean 24-hour PWV was 7 (SD, 0.8) m/second. Based on the data accrued, the study had greater than 98% power to detect a 5-mm Hg difference in systolic BP (0.4 SDs), a 4-mm Hg difference in diastolic BP (0.5 SDs), and a 0.4-m/second difference in PWV (0.5 SDs) between children with SCT and those without SCT.

Table 1 displays the characteristics of the participants according to SCT carrier status. The mean 24-hour systolic and diastolic BPs in subjects with SCT were 116 (SD, 11.5) mm Hg and 64 (SD, 7) mm Hg, respectively. In subjects without SCT, the corresponding 24-hour BP values were 117 (SD, 11.4) mm Hg and 64 (SD, 6.8) mm Hg, respectively (*P* = 0.8551 and *P* = 0.9691, respectively, for comparison between SCT carriers and noncarriers). There were no statistically significant between-group differences in eGFR, clinic BP, clinic PWV, or 24-hour PWV. There were no between-group differences in the prevalence of masked hypertension, white-coat hypertension, or nondipping status. Urinary sodium and potassium concentrations were 19.5 mmol/L (95% CI: 1.4, 37.6; *P* = 0.0352) and 13.5 mmol/L (CI: 5.8, 21.2; *P* = 0. 0006) lower in SCT carriers than in noncarriers.

**Table 1.** Characteristics of Adolescent Study Subjects (Ages 11–17 Years) According to Sickle Cell Trait Carrier Status, Nairobi, Kenya, 2015–2016

Characteristic	SCT Noncarriers (*n* = 567)			SCT Carriers (*n* = 103)			*P* Value
	No. of Children	%	Mean (SD)	No. of Children	%	Mean (SD)	
Female sex	323	57		49	48		0.08
Complete ABPM data	516	91		93	90		0.82
White-coat hypertension^a^	23	44		3	3		0.59
Masked hypertension^a^	37	7		8	9		0.63
Nondipping BP pattern^a^	17	3		2	2		0.55
Age, years			13.2 (2.2)			13.8 (2.3)	0.09
Body mass index^b^			18.8 (3.1)			19.0 (3.1)	0.63
Mid-upper arm circumference, cm			23.2 (3.6)			23.5 (3.8)	0.41
Hemoglobin concentration, mg/dL			13.2 (1.5)			13.4 (1.6)	0.15
White blood cell count, cells × 10^9^/L			5.5 (1.5)			5.6 (1.3)	0.78
Platelet count, cells × 10^9^/L			310.4 (86.5)			305.8 (95.3)	0.64
Serum sodium level, mmol/L			139.1 (6.3)			139.1 (5.6)	0.94
Serum potassium level, mmol/L			4.9 (0.6)			5.0 (0.8)	0.09
Socioeconomic status (MDPI score^c^)			2.2 (1.3)			2.1 (1.3)	0.55
Clinic BP, mm Hg							
Systolic			98.2 (10.8)			99.3 (12.6)	0.31
Diastolic			63.6 (8.1)			64.4 (8.9)	0.32
Pulse wave velocity, m/second			7.0 (0.8)			7.1 (0.8)	0.26
eGFR, mL/minute/1.73 m^2^			110.3 (14.0)			107.8 (14.2)	0.12
Urinary albumin:creatinine ratio			3.6 (17.5)			3.5 (7.5)	0.96
Urinary sodium level, mmol/L			135.7 (73.1)			119.1 (45.4)	0.03
Urinary potassium level, mmol/L			48.4 (30.5)			36.2 (23.2)	0.0001

Abbreviations: ABPM, ambulatory blood pressure monitoring; BP, blood pressure; eGFR, estimated glomerular filtration rate; MDPI, multidimensional poverty index; SCT, sickle cell trait; SD, standard deviation.

^a^ Data on white-coat hypertension, masked hypertension, and nondipping pattern were based on the 609 children with complete ABPM data.

^b^ Weight (kg)/height (m)^2^.

^c^ A household is considered poor if the MDPI score is greater than 3.

**Figure 2.**
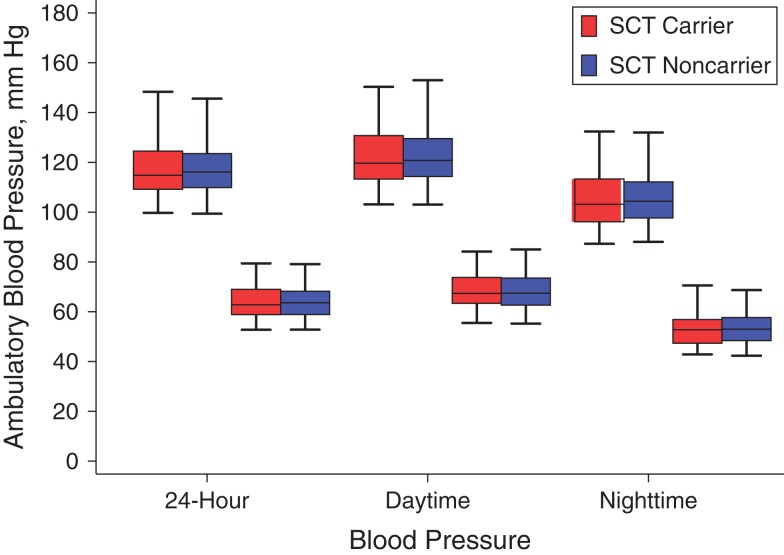
Ambulatory blood pressure monitoring measurements among adolescents aged 11–17 years according to sickle cell trait (SCT) carrier status, Nairobi, Kenya, 2015–2016. For each set of 24-hour, daytime (08:00–20:00 hours (8 am–8 pm)), and nighttime (00:00–06:00 hours (12 am–6 am)) measures, the plots on the left are for systolic blood pressure and those on the right are for diastolic blood pressure. Bars, 95% confidence intervals.

Figure 2 displays the distributions of 24-hour, daytime, and nighttime BPs in study participants by SCT carrier status. All measures were similar for SCT carriers and noncarriers.

Table 2 displays the results of regression analyses examining whether SCT influenced 24-hour systolic and diastolic BP after adjustment for age, sex, body mass index, eGFR, and PWV. While age, sex, body mass index, and eGFR were all associated with 24-hour systolic BP, there was no association between SCT and 24-hour BP measures. PWV displayed the strongest association with both systolic and diastolic BP. Additional adjustment for urinary sodium and potassium levels made no material difference in the results.

**Table 2.** Predictors of Mean 24-Hour Systolic and Diastolic Blood Pressures Among Adolescents Aged 11–17 Years, Nairobi, Kenya, 2015–2016^a^

Characteristic	24-Hour SBP			24-Hour DBP		
	β	95% CI	*P* Value	β	95% CI	*P* Value
Age, years	0.60	0.10, 1.10	0.03	0.01	–0.30, 0.30	0.96
Male sex	2.40	0.40, 4.30	0.02	–0.06	–1.30, 1.20	0.92
Body mass index^b^	0.60	0.20, 1.00	0.001	0.20	–0.09, 0.40	0.21
eGFR, mL/minute/1.73 m^2^	0.08	0.01, 0.15	0.02	0.01	–0.03, 0.05	0.58
Pulse wave velocity, m/second	2.80	1.60, 4.10	<0.001	2.70	1.90, 3.50	<0.001
Sickle cell trait carrier status	0.10	–2.40, 2.60	0.92	0.40	–1.10, 2.00	0.58

Abbreviations: CI, confidence interval; DBP, diastolic blood pressure; eGFR, estimated glomerular filtration rate; SBP, systolic blood pressure.

^a^ Multivariate analyses were conducted using data from participants who had complete ambulatory blood pressure monitoring data (*n* = 609).

^b^ Weight (kg)/height (m)^2^.

## DISCUSSION

It has previously been hypothesized that the excess risk of cardiovascular disease observed in African populations may result from pleiotropic effects of genetic polymorphisms that protect them from common infections during childhood. An elegant example of this is variants of the apolipoprotein L1 gene (*APOL1*), which, while reducing the risk of trypanosomiasis, increase the risk of hypertension-associated chronic kidney disease (33). Malaria has exerted the strongest known selective pressure on the human genome, SCT being prominent among the polymorphisms under positive selection (34). Given the previously documented excess cardiovascular and renal events observed in both sickle cell disease and SCT, we hypothesized that persons with SCT would have different BPs than those without SCT.

In this detailed study of BP phenotypes and arterial stiffness among adolescents who were selected because they had had little exposure to malaria throughout childhood, we did not find any differences between those with and without SCT. Because the exposure measurement was a genetic trait acquired at conception and the participants were ascertained to have remained in the same malaria-free environment since birth, we believe that this study suggests that a direct effect of SCT on BP and indices of arterial stiffness is highly improbable within the first 11–17 years of life.

Urinary albumin:creatinine ratio and eGFR were the same in SCT carriers and noncarriers in this study. In a study conducted among blacks in the United States showing increased renal events in SCT carriers, most participants were recruited at 45 years of age and above (8), much older than the population we recruited in this study. However, we found significantly lower urinary electrolyte levels in SCT carriers, which could be attributed to the hyposthenuria (impaired urine-concentrating ability) that has previously been described in SCT (35).

While we failed to detect any meaningful effect of SCT on BP and arterial stiffness, the results of this study do have important implications. It seems unlikely that increased BP and arterial stiffness precede or are involved in the pathogenesis of cardiovascular and renal events in persons with SCT. In view of this, studies of other biomarkers that could predict the development of chronic kidney disease in persons with SCT are warranted. Because persons with SCT form a significant proportion of the population in many developing countries, as well as in developed countries, early identification of risk factors in this subgroup of individuals could have significant population-wide benefits.

An alternative to the hypothesis that genetic variations protective against infectious diseases predispose people to cardiovascular disease is that in some instances the infectious diseases themselves may have long-term consequences in survivors, including the development of hypertension (14). One robust way to test such hypotheses is by utilizing Mendelian randomization techniques in which BP is compared in persons with and without genetic variants that are associated with the infectious disease. An important prerequisite for using these variants is that they should not affect the outcome (BP) in the absence of the infectious disease (malaria). The results of this study suggest that SCT does not influence BP in the absence of malaria and can therefore be used as an instrumental variable in Mendelian randomization studies to test the malaria–high BP hypothesis (14). SCT is a particularly attractive candidate for such studies, as it is relatively common in areas with malaria and displays a very strong protective effect against both mild and severe malaria (2, 4), thus reducing sample size requirements for such studies. Confirmation of the hypothesis would represent a paradigm shift in understanding the pathogenesis of hypertension in many developing-country settings where malaria is endemic (36).

A major strength of this population-based study was the use of ABPM, which is considered the reference standard for BP measurement in children (16). The study had enough power to detect very small differences in BP and PWV. We also used health and demographic surveillance system records that were prospectively collected in order to ascertain residence in a nonmalaria zone, there being no better method of doing this in sub-Saharan Africa.

A potential limitation of this study was the limited age range of subjects recruited, necessitated by the fact that there were no long-term residency records for older individuals. Most demographic surveillance systems in Africa were established between the late 1990s and the early 2000s (37). Recruiting older persons would have compromised data on residency status in childhood, the period when malaria risk is highest. In addition, older subjects would be more likely to have acquired additional risk factors for hypertension, including chronic kidney disease, that would have confounded the analyses. While BP differences are likely to be larger at older ages, it is known that differences in adult BP emerge in childhood (38, 39) and that childhood BP levels are predictive of adult BP (40). Therefore, the absence of even a small difference in carefully measured BP and arterial stiffness in our study of adolescents suggests that it is very unlikely such differences would emerge in the future.

Although the study had sufficient power to detect differences in continuous variables such as mean nighttime and daytime BP, it lacked power to detect differences in the prevalences of categorical variables such as masked hypertension and white-coat hypertension. This could form the basis of future studies.

An additional limitation of this study is the fact that, as with many BP measurement devices, validation studies for the Arteriograph24 have only been done in adults (25). It is unlikely, however, that measurement error significantly influenced the result, as the oscillometric principle used by the device has been validated in children and is particularly suited for pediatric ambulatory BP studies (16, 41).

Nonresponders in this study were slightly older than those who participated, but the 0.6-year difference is unlikely to have significantly biased our results. We also observed no significant differences between children who had acceptable ABPM data and those who did not, suggesting that the data presented are representative of the larger population of persons with SCT.

In summary, we have demonstrated that the presence of SCT does not influence BP and arterial stiffness in Kenyan adolescents.

## Abbreviations

ABPM: ambulatory blood pressure monitoring
BP: blood pressure
CI: confidence interval
eGFR: estimated glomerular filtration rate
PWV: pulse wave velocity
SCT: sickle cell trait
SD: standard deviation
